# Fresh-marketable tomato yields enhanced by moderate weed control and suppressed fruit dehiscence with woodchip mulching

**DOI:** 10.1038/s41598-022-15568-x

**Published:** 2022-08-02

**Authors:** Sakae Horimoto, Kazuaki Fukuda, Jin Yoshimura, Atsushi Ishida

**Affiliations:** 1 Department of Applied Biology, Kyoto Institute of Technology, Ukyo, Kyoto 616-8354 Japan; 2 Institute of Tropical Medicine, Nagasaki University, Sakamoto, Nagasaki, Nagasaki 852-8523 Japan; 3 Faculty of Science, Tokyo Metropolitan University, Minami-Osawa, Hachioji, Tokyo 192-0397 Japan; 4 The University Museum, The University of Tokyo, Hongo, Bunkyo, Tokyo 113-0033 Japan; 5 Center for Ecological Research, Kyoto University, Otsu, Shiga 520-2113 Japan

**Keywords:** Plant sciences, Ecology

## Abstract

The use of plastic film imposes various environmental risks in agroecosystems. The replacement of plastics with organic materials for mulching has been suggested to enhance the sustainability of agroecosystems. However, whether woodchip mulch can be used for annual crops needs to be verified. We examined the effects of mulberry woodchip mulches on tomato-fruit yields over two successive years. Mulberry is the unique food plant of silkworms, and it will be better if its pruned shoots can be recycled rather than incinerated as waste. Setting three treatments, including woodchip mulch, weed-free and weedy (i.e., unweeded) treatments, we compared the amounts of fresh-marketable and unmarketable tomato fruits. The yields of fresh-marketable tomato fruits in the woodchip mulch treatment were significantly 16–57% higher than those in the weed-free treatment and comparable to those in the weedy treatment. The yields of unmarketable dehiscent tomato fruits in the weed-free treatment were significantly 46–86% higher than those of the other two treatments. The woodchip mulches extensively suppressed the weed density, while the grown weeds became large, preventing strong sunlight exposure and dehiscence of tomato fruits. Current results suggest that woodchips could be a possible alternative to plastics, facilitating climate change mitigation with agroforestry practices.

## Introduction

The plastics have been widely used for many products in modern societies, e.g., packaging material, furniture, and agricultural applications (such as greenhouses and mulches). Recently, plastics have been recognized as a serious waste problem because they do not fully decay. The replacement of plastics by natural materials has been initiated in various fields. Even in agricultural applications, plastics need to be replaced by natural organic materials^1^. Black plastic mulches have been widely employed worldwide to cover agricultural surfaces because of their economic affordability^2–7^. Covering of crop fields with mulches is required to obtain a physical barrier to suppress soil water evaporation and erosion, control weeds, maintain good soil structure and protect crops from soil contamination, resulting in high crop yields^8–11^. Although herbicides, such as glyphosate, are widely used for control weeds in the world, glyphosate-resistant weed populations have been also reported^12,13^. Thus, alternative herbicides^14,15^ and new non-chemical weed control methods^16^ have intensively been tested.

The use of plastic films in horticultural crop applications has remarkably increased since 1990, and approximately 700 gigatons of plastic sheet are being used annually as mulching materials worldwide^17^. Although plastic materials are often superior to organic materials for crop mulching, the use of plastic film imposes several serious environmental hazards on agroecosystems. For example, (1) the manufacturing process of polyethene materials requires the use of fossil fuels, and plastics induce waste problems; (2) the release of CO_2_ gas and toxic ingredients in air occurs when the plastic materials are burned on farmlands after cropping seasons; and (3) small plastic debris remains in crop fields and then disperses widely into natural ecosystems, occasionally flowing into sea water^18–21^. Moreover, microplastics in the sea have become serious threats to fishes, sea birds, and other oceanic animals^22,23^.

As alternatives to plastic mulches, the use of organic materials has been recently promoted to enhance the sustainability of agroecosystems^1,24^. Although organic materials are frequently unaffordable, the use of dead plants and cover plants for organic mulches provides a significant environmental advantage^11^. Even with organic mulches, the soil water contents and temperature can be maintained at adequate constant levels by reducing evaporation^25,26^. Furthermore, dead plant parts, cover crops and other organic matter are well mixed in the soil after the cropping season, increasing the soil nutrient levels^27–30^. Ultimately, we can avoid the waste problems from nondecaying plastic films, allowing for climate change mitigation and adaptation to meet challenges^24^.

In agroforestry, the effective use of pruned woody shoots is generally desired. Currently, woodchip mulches are applied to urban gardens, fruit farms and greenbelts along roadsides globally^9,26^. In silviculture, woodchip mulches are known to improve the survival and establishment of planted tree seedlings^31^. However, the application of this mulch for annual crops is still rare in agriculture^1,32,33^. A successive two-year application to a bean (*Lens culinaris*) planting area showed that weed control was successful, and the grain yields were not different, implying that substitution with organic mulch is a possible option^33^. However, the extracts from woodchips, which include many chemical compounds, occasionally shows allelopathic effects on plant growth and seed germination^34–36^. The usefulness of woodchip mulches for annual crop production is thus still debatable^37^. Therefore, we should carefully examine the positive and negative effects of woodchip mulches applied to crop fields over a long-term period^1,11,33^.

Silkworm rearing has been an important industry worldwide since the last twentieth century. In Japan, industrial silk production has decreased during the last 50 years. However, silkworm rearing in Kyoto and the surrounding areas is still highly prosperous because of the industrial production of silk clothing, such as the Kimono, a traditional Japanese cloth. Furthermore, in China and India, the silkworm rearing has recently become one of the main industries^38^. Mulberry (*Morus alba* L.), which is the unique food plant of silkworms, is a winter-deciduous, fast-growing tree species. Large amounts of pruned shoots are produced every year for silkworm rearing. Nevertheless, pruned mulberry shoots are mostly burned on farmlands because of their long composting time and the associated costs and the low demand for composts in Japan. The burning of large amounts of organic materials not only causes a rapid increase in air CO_2_ concentrations but also possibly poses health risks to residents in burning areas because of the resultant heavy haze^39,40^. Therefore, the reuse of pruned plant materials in agroforestry is one resolution to these important global environmental issues^24^.

**Figure 1:**
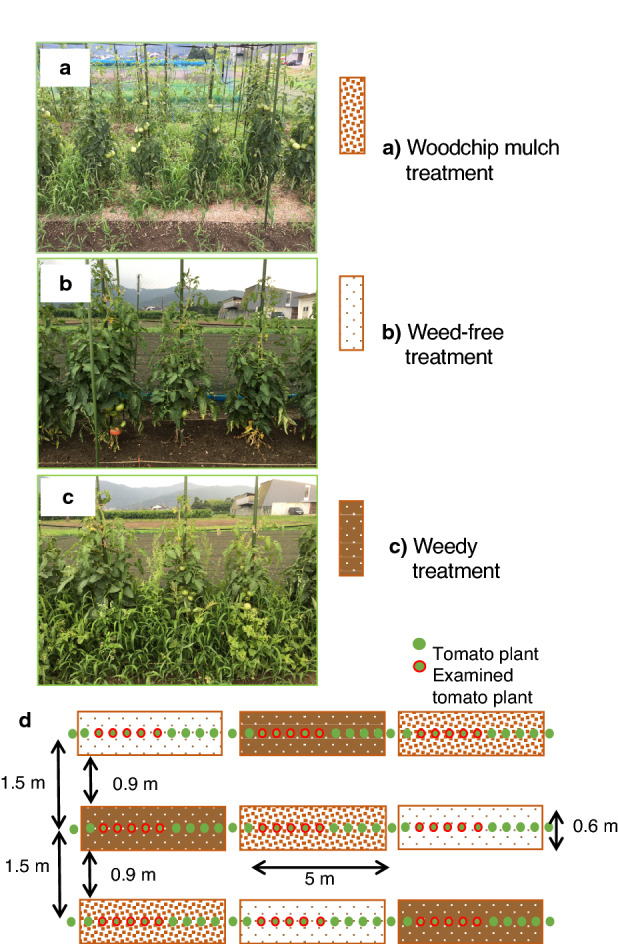
Photos of the examined cultivation treatments and the planting design for tomato plants. (**a**) The woodchip mulch treatment, (**b**) the weed-free (i.e., weeded) treatment, (**c**) the weedy (i.e., unweeded) treatment and (**d**) the cultivation design. Each treatment had three replicates (separated ridges). Tomato seedlings were transplanted at a 0.5 m interspace on 26 April 2016 and on 9 May 2017. The photos were taken by K. Fukuda (the second author).

**Figure 2:**
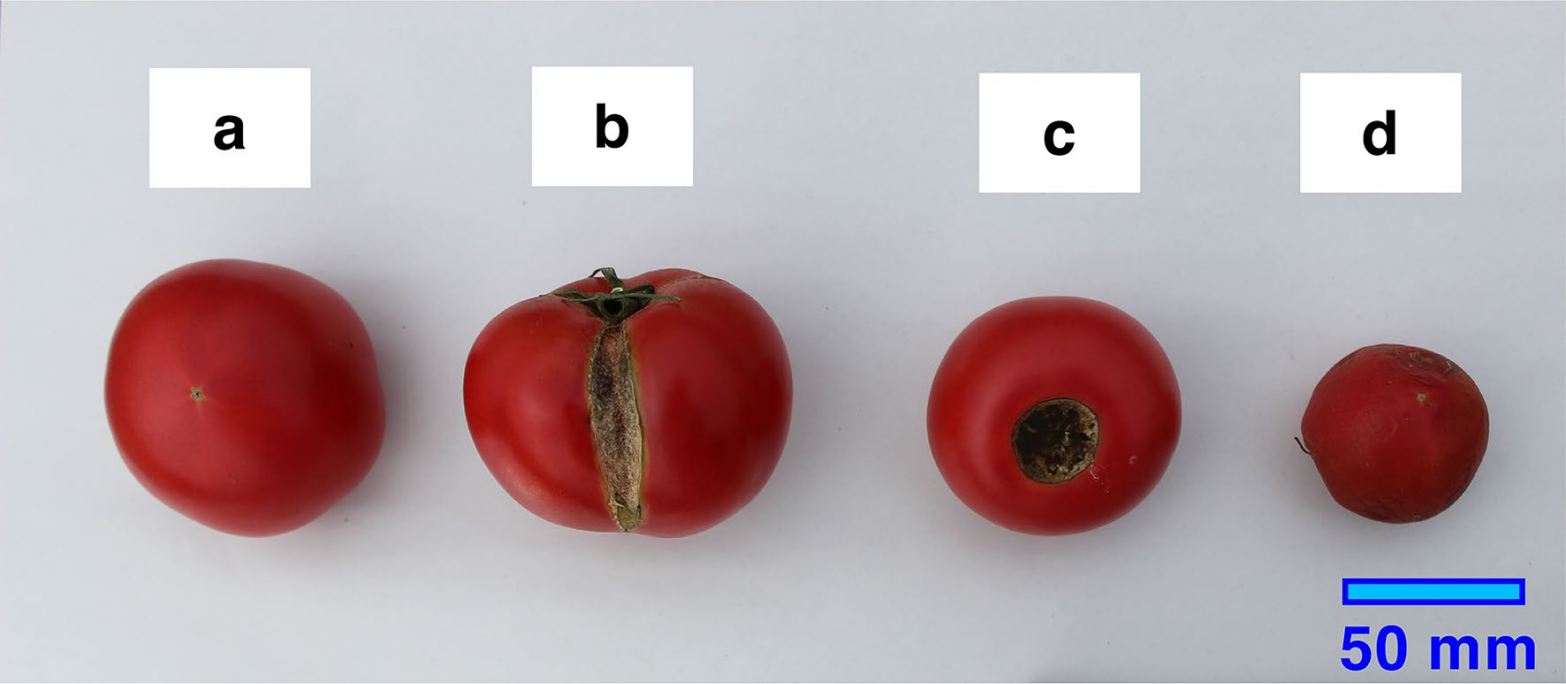
The separate types of tomato fruits harvested. (**a**) A fresh-marketable fruit, (**b**) a dehiscent (cracking) fruit, (**c**) a fruit infected by blossom-end rot and (**d**) a fruit that was too small (less than 115 g in fresh mass). (**b–d**) Typical types of unmarketable tomato fruits in Japan. The photos were taken by S. Horimoto (the first author).

Using pruned mulberry shoots, we examined the effects of woodchip mulches during two successive years on the fruit yields of an annual crop, tomato (*Solanum lycopersicum* L.), in Japan. To evaluate the effects of weed control by the woodchip mulches on tomato fruit yields, we set three treatments as follows: (1) woodchip mulch, (2) weed-free (i.e., weeded) and (3) weedy (i.e., unweeded) treatments. Each treatment was conducted in three replicates (ridges), and we transplanted tomato seedlings into these ridges in spring of 2016 and 2017 (Fig. 1). In this study, the soil nutrient levels were fully maintained to remove the fertilization effects of woodchip mulching on tomato fruit production. The tomato fruits harvested from three treatments were divided into (a) fresh-marketable fruits and the following three types of unmarketable fruits: (b) dehiscent fruits, (c) fruits infected by blossom-end rot and (d) fruits that were too small (Fig. 2). Here, we show that the woodchip mulches sufficiently suppress weed plant density (the weed density in the woodchip mulch treatment was 2–11% of that in the weedy treatment), but the weeds that do occur become large and tall in size (the weed mass per land area in the woodchip mulch treatment was 68–104% of that in the weedy treatment), reducing the dehiscence (cracking) of tomato fruits by shading. As a result, the mulberry woodchip mulches enhance fresh-marketable tomato yields compared with the weed-free treatments, which have high labor costs. This result shows that as a replacement of plastic mulches, woodchip mulches are useful even for annual crop production, indicating that agroforestry should be part of global climate change mitigation and adaptation.

## Results

### Microclimate

**Figure 3:**
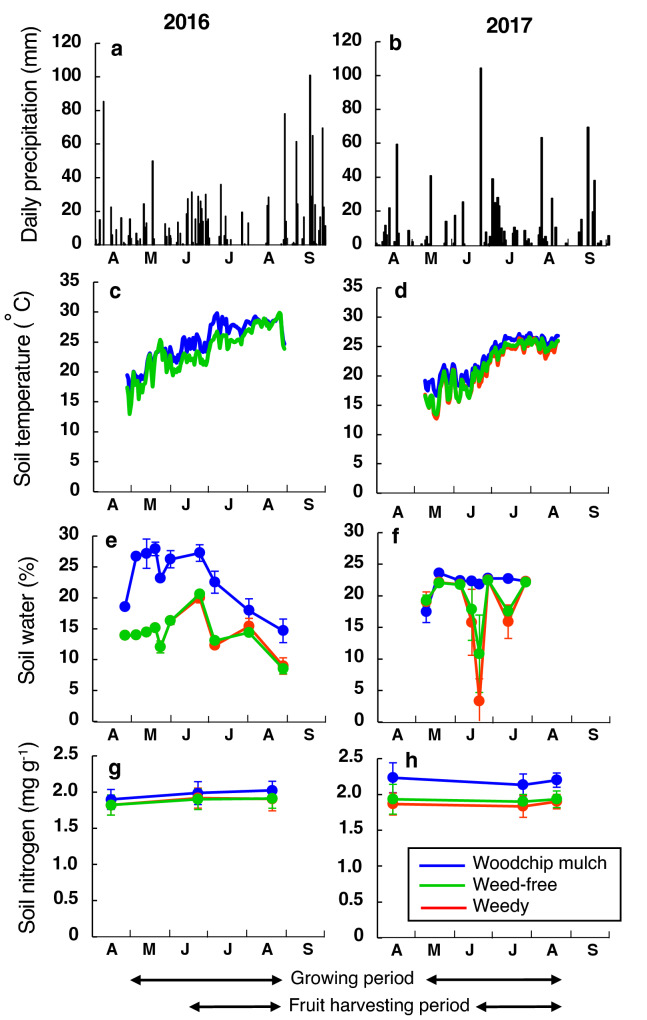
The seasonal changes in the microclimate. (**a**,**b**) Daily precipitation, (**c**,**d**) soil temperatures at 5 cm below the ground surface, (**e**,**f**) water contents in the soil from 0 to 10 cm below the ground surface (volume percentage), and (**g**,**h**) dry mass-based soil nitrogen concentrations from April to September in (**a**,**c**,**e**,**g**) 2016 and (**b**,**d**,**f**,**h**) 2017. The mean values in the woodchip mulch (blue), the weed-free (red) and the weedy (green) treatments are shown, and the error bars for soil water and nitrogen show ± 1 S.D. The growing period and fruit harvesting period in each year are shown by double-headed arrows.

During the last three decades (1991–2020) in Kyoto, the average yearly precipitation and air temperature were 1522.9 mm and 16.2 °C, respectively (Kyoto Climate Observatory). From May to August (the tomato growing season) during these decades, the mean precipitation and air temperature were 728.5 mm and 24.7 °C, respectively. The precipitation from May to August in 2016 and 2017 was 682.5 mm and 551.0 mm, respectively (Fig. 3a,b). Compared with the average value during the last three decades, the precipitation during the growing seasons in the present study was thus low, especially in 2017.

From May to August, the average soil temperatures at 5 cm below the ground surface in 2016 were 25.3 °C and 23.8 °C in the woodchip mulch and the weedy treatments, respectively; those in 2017 were 23.1 °C, 21.4 °C and 21.7 °C in the woodchip mulch, the weed-free treatment and the weedy treatment, respectively (Fig. 3c,d). The soil temperatures in the woodchip mulch treatment were higher than those in the other treatments in both years.

Irrigation was done only once just after tomato planting in all treatments. The soil water contents during the growing seasons in 2016 were always higher in the woodchip mulch than in the other treatments. In contrast, no conspicuous differences were found in the maximum soil water contents in 2017. In the weed-free and the weedy treatments, the soil water contents decreased markedly in the mid-June and mid-July 2017, when periods of no precipitation lasted for 12 and 5 days, respectively. However, even in these short drought periods in 2017, conspicuous decreases in soil water contents were not detected in the woodchip mulch treatment (Fig. 3e,f). Therefore, the woodchip mulches effectively suppressed excess soil evaporation, preventing a temporary extreme drop in soil water contents. The seasonal change in soil nitrogen (N) concentrations in 2016 ranged from 1.88 to 1.97 mg g^−1^ (Fig. 3g,h), and no significant differences among the treatments were found in 2016 (Supplementary Table 1). Comparing the two years, in only the woodchip mulch treatment, the soil N concentrations increased significantly from 2016 to 2017, showing the delayed effects of woodchip mulching on soil N accumulated from the dead plant materials.

### Weed control

In the experimental field, three families of Amaranthaceae, Asteraceae and Poaceae were predominant weed plants (Supplementary Table 2). These families consist of annual plants, and they are key weeds. In the weed dry mass, *Digitaria ciliaris* (Retz.) Koeler (Poaceae) accounted for the highest weed dry mass in all treatments (Supplementary Table A3); the maximum heights of their flower stalks reached approximately 70 cm high. The weed density was significantly higher in the weedy treatment (190.7–316.7 m^−2^) than in the woodchip mulch treatment (7.2–9.8 m^−2^) (Table 1), showing that the woodchip mulches effectively decreased the number of weeds (i.e., weed density) even though we did not apply other weed control. At the end of harvesting in the cropping season (late August), the weed dry mass per land area in 2016 was significantly higher in the weedy treatment (723.3 g m^−2^) than in the woodchip mulch treatment (469.9 g m^−2^), whereas no significant differences were found between these treatments in 2017 (919.5–954.1 g m^−2^). Consequently, the aboveground dry mass/weed density ratios in the woodchip mulch treatment were 47.9 and 34.7 in 2016 and 2017, respectively, whereas those in the weedy treatment were 3.8 and 2.3 in 2016 and 2017, respectively. These data showed that the weed density in the woodchip mulch treatment was 2–11% of that in the weedy treatment, and the weed mass per land area in the woodchip mulch treatment was 68–104% of that in the weedy treatment. These data indicate that the averaged aboveground biomass of one weed plant was approximately 10 times that in the no weed (i.e., weedy) treatment, resulting from more active tillering in the woodchip mulch treatment having low weed density compared with the weedy treatment having high weed density (see Fig. 1a).

**Table 1:** Mean (1 SD) values of weed density and the aboveground dry mass of weeds among three replicates in each treatment.

Year	2016				2017			
Date	14 June	02 August	23 August	23 August	20 June	25 July	21 August	21 August
(Unit)	Weed density (plant m^−2^)		Weed mass (g m^−2^)		Weed density (plant m^−2^)		Weed mass (g m^−2^)	
Woodchip mulch	7.2 **a**	9.8 **a**	9.8 **a**	467.9 **a**	31.1 **a**	29.2 **a**	27.5 **a**	954.1 **a**
	(0.7)	(0.8)	(1.1)	(57.7)	(6.3)	(4.6)	(8.5)	(114.3)
Weed-free	325.9 **b**	480.6 **b**	0	0	767.6 **b**	157.1 **b**	95.2 **a**	13.9 **b**
	(21.6)	(211.3)	(0)	(0)	(290.4)	(84.4)	(19.4)	(4.0)
Weedy	316.7 **b**	191.7 **c**	190.7 **b**	723.3 **b**	497.1 **c**	260.0 **c**	392.4 **b**	919.5 **a**
	(53.6)	(31.5)	(104.4)	(592.4)	(235.4)	(104.7)	(153.4)	(111.8)
AIC	116.3	147.7	99.7	172.3	161.2	136.2	139.4	136.6

To examine the weed dry mass, all weeds were harvested on 23 August 2016 and on 21 August 2017 in all treatments. In the weed-free treatment, all weeds were harvested three times a year by hand. Different letters (a, b, c) on each day show significant differences (*P* < 0.05) among the treatments by generalized linear mixed models (GLMMs) including random effects among three replicates.

### Tomato fruit production per plant

**Figure 4:**
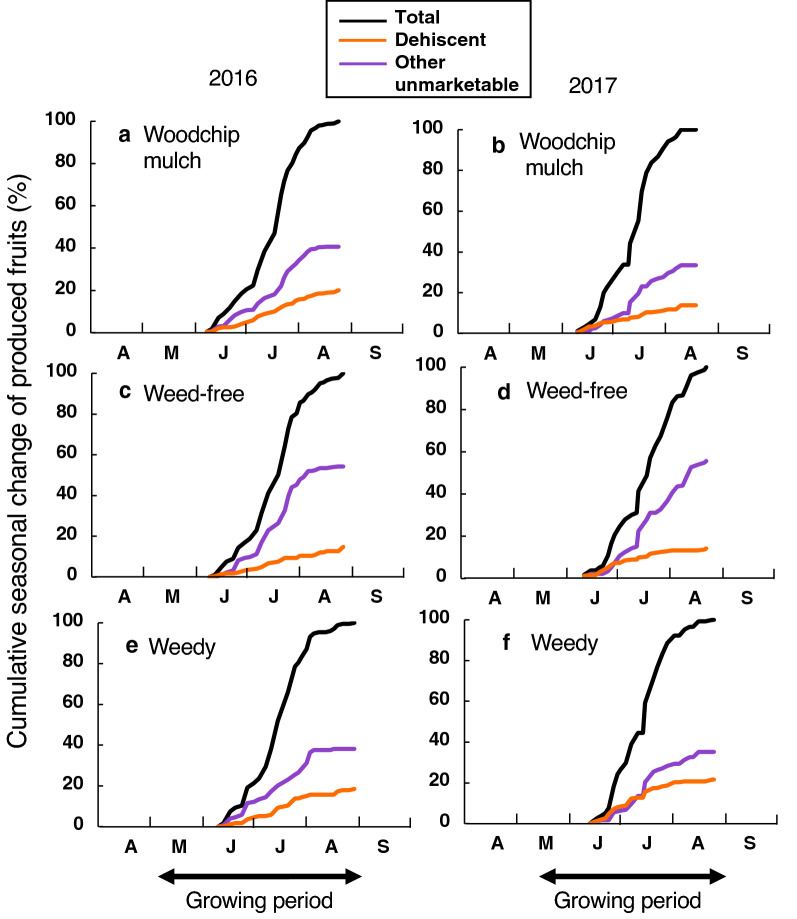
Cumulative seasonal changes in fruit production. The cumulative number of total fruits (black), the number of dehiscent fruits (orange) and the number of other unmarketable fruits (purple) during the fruit harvesting periods in (**a**,**b**) the woodchip mulch, (**c**,**d**) the weed free and (**e**,**f**) the weedy treatments (**a**,**c**,**e**) in 2016 and (**b**,**d**,**f**) in 2017. Fruit harvesting started on 11 June 2016 and on 12 June 2017. The growing period in each year is shown by a double-headed arrow. See Fig. 2 for harvested fruit types.

Fruit harvesting was conducted at approximately three-days intervals from mid-June to late August. The highest amounts were harvested in July (Fig. 4). The number of unmarketable tomato fruits reached 56.6–69.1% and 47.3–69.3% out of the total number of harvested fruits in 2016 and 2017, respectively. The number of dehiscent fruits reached 66.9–78.6% and 61.9–79.6% out of total unmarketable fruits in 2016 and 2017, respectively; numbers of fruits infected by blossom-end rot reached 1.0–1.9% and 1.9–10.4% out of the total unmarketable fruits in 2016 and 2017, respectively; numbers of too small fruits reached 18.2–26.2% and 27.2–45.5% out of the total unmarketable fruits in 2016 and 2017, respectively. Thus, the dehiscence of tomato fruits was the most serious factor making fruits unmarketable. Almost all unmarketable tomato fruits (Fig. 2b–d) are ploughed into the soil in Japan.

**Figure 5:**
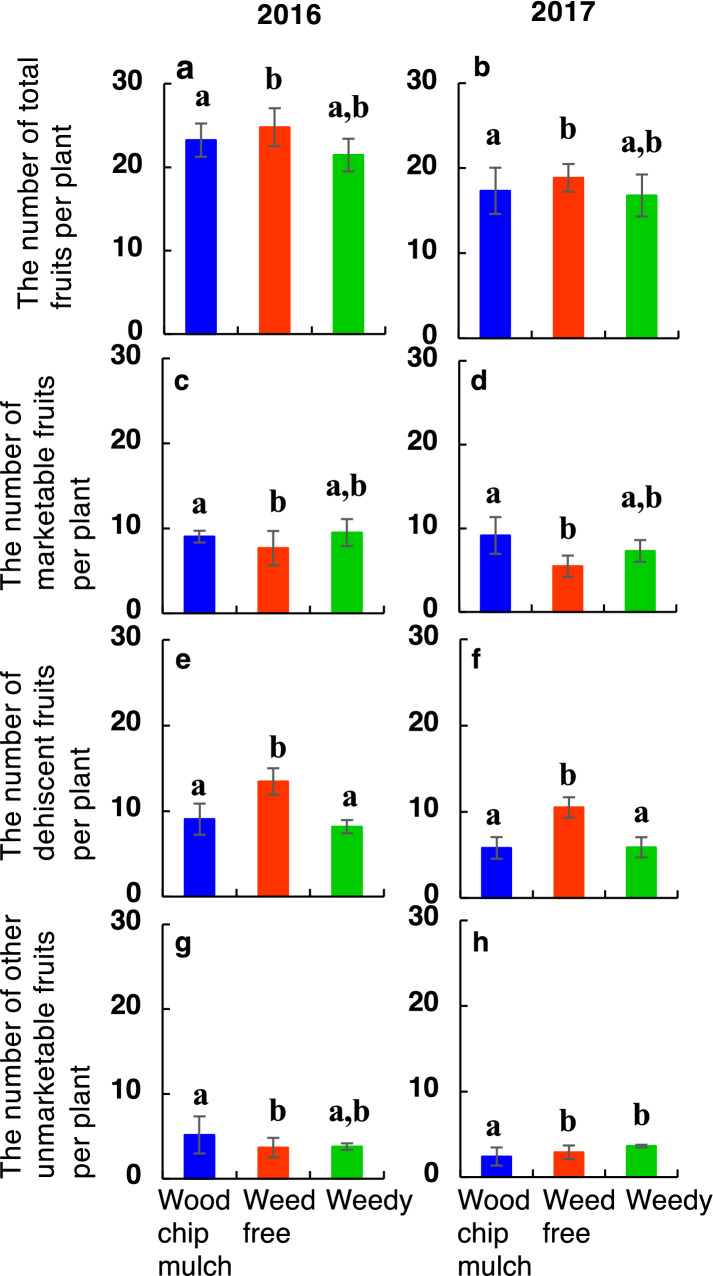
The total number of harvested tomato fruits per plant. (**a**,**b**) Total fruits, (**c**,**d**) fresh-marketable fruits, (**e**,**f**) dehiscent fruits and (**g**,**h**) the other unmarketable fruits per plant (**a**,**c**,**e**,**g**) in 2016 and (**b**,**d**,**f**,**h**) in 2017. The mean values in the woodchip mulch (blue), weed-free (red) and weedy (green) treatments are shown. Error bars show ± 1 S.D. Different letters just above the vertical vars in each panel show significant differences (*P* < 0.05) among the treatments in each year. See Fig. 2 for harvested fruit types.

There were no significant differences in the total number of harvested tomato fruits per plant (21.6–22.7 in 2016, 16.9–19.3 in 2017) among the treatments in each year (Fig. 5, Table 2). However, the number of dehiscent fruits per plant was significantly 41–61% higher in the weed-free treatment (13.4 in 2016, 10.8 in 2017) than in the other two treatments (8.3–9.2 in 2016, 5.8–5.9 in 2017) in both years. Because of the increased fruit dehiscence, the number of fresh-marketable fruits per plant was significantly lower in the weed-free treatment (7.7 in 2016, 5.8 in 2017) than in the other two treatments (8.9–9.4 in 2016, 7.3–9.1 in 2017) in both years.

**Table 2:** Mean (1 SD) values of the number of tomato fruits per plant among three replicates in each treatment in 2016 and 2017.

	Total fruits	Marketable fruits	Dehiscent fruits	Other unmarketable fruits
**Year: 2016**				
Wood chip mulch	22.7 **a**	8.9 **a**	9.2 **a**	4.6 **a**
	(3.9)	(3.5)	(3.4)	(3.9)
Weed-free	24.8 **b**	7.7 **b**	13.4 **b**	3.7 **b**
	(3.4)	(3.5)	(3.4)	(1.7)
Weedy	21.6 **a,b**	9.4 **a,b**	8.3 **a**	4.0 **a,b**
	(4.7)	(3.1)	(2.1)	(2.1)
AIC	211.6	194.4	193.5	188.2
**Year: 2017**				
Wood chip mulch	17.3 **a**	9.1 **a**	5.8 **a**	2.4 **a**
	(3.4)	(3.1)	(2.2)	(1.3)
Weed-free	19.3 **b**	5.8 **b**	10.8 **b**	2.8 **b**
	(4.1)	(2.2)	(3.4)	(1.8)
Weedy	16.9 **a,b**	7.3 **a,b**	5.9 **a**	3.6 **b**
	(3.0)	(1.8)	(2.7)	(1.6)
AIC	211.6	194.4	209.1	162

Other unmarketable fruits include fruits that too small fruits and pathogen-infected fruits (see Fig. 2). Different letters (a, b, c) in each fruit type show significant differences (*P* < 0.05) among the treatments by generalized linear mixed models (GLMMs) including random effects among three replicates.

## Discussion

The present study shows that woodchip mulches are useful for the fruit production of tomato, an annual crop. Fruit dehiscence (cracking) is one of the main problems in tomato cultivation^41,42^ due to excess watering^42^, increasing fruit turgor^43^ and strong sunlight exposure^44^. In the present study also showed that fruit dehiscence was the most serious factor (54–56% out of the total tomato fruits in the weed-free treatment), increasing the number of unmarketable fruits (Table 2). Strong sunlight exposure at the fruit-maturing stage was likely to lead to the dehiscence of tomato fruits. Furthermore, the mulberry woodchip mulches did not show any negative chemical effects, such as allelopathic activity, on plant growth and fruit yields. The woodchip mulches significantly suppressed weed density, but the successfully growing weeds developed into large plants, probably because of the low plant density (see Fig. 1a). Taller weeds would provide a moderate shade for the tomato fruits, and as a result, many fruits were able to successfully escape dehiscence damages. These mechanisms contributed to the significant increases in the proportion of fresh-marketable fruits in the woodchip mulching treatment. In another experimental study, organic mulches from various materials were applied to tomato plantings over three successive years^1^. In their pioneering study, more marketable fruits were occasionally produced with organic mulches than with black plastic mulches; however, the significant differences disappeared depending on year and the type of organic materials. Their study indicates that the selection of organic materials, adjusted to the environmental conditions of farmlands, is an important factor for optimizing tomato fruit production^1^.

Effective weed control by organic mulches has been recognized globally. Organic mulches applied under deep soil cover show higher weed control ability than those applied under shallow soil cover^45^. To obtain sufficient successful weed control in tomato fruit production, a minimum thickness of 10–15 cm of woodchip mulches is required for gravel mulch topping effects^46^. In the current study, we adopted a woodchip top-covering of only approximately 3 cm in thickness, which is very thin compared with 10–15 cm. Even with this level of thinness, the weed density was effectively suppressed (Table 1), and the production of fresh-marketable tomato fruits was significantly higher than that of the weed-free treatment in both years (Fig. 5, Table 2). For fresh-marketable tomato production, a top covering with a 10–15 cm thickness is probably too thick because it would inhibit the germination and growth of nearly all weeds; in this study, a few weeds with a large plant size in the thin (3 cm) woodchip mulch treatment would contribute to provide adequate shading to suppress fruit dehiscence. Because weeding (i.e., the weed-free treatment) produced more dehiscent tomato fruits, probably due to strong sunlight exposure (Fig. 5, Table 2), the moderate weed control by thin-layered woodchip mulching was useful for enhancing fresh-marketable tomato fruit production. The cultivars for fresh-marketable tomato fruits usually have thin skin for fresh eating, especially in Japan; therefore, fruit dehiscence occurs easily due to strong ultraviolet exposure^44^ and excessive watering^43^. The weed density reduction by the woodchip mulching leads to active tillering of growing weeds (Fig. 1a), adding adequate shading when the tomato fruits are maturing. Thus, in the farmlands with many annual weeds, the total amounts of woodchips needed will be much less than expected. An greenhouse experiment in tomato showed that either shade covering in each fruit cluster or overall 20%-cut shading in the greenhouse suppressed effectively the occurring of fruit dehiscence^44^. In the farmlands of more expensive fruits such as fresh-marketable grape and peach, we usually cover each fruit with waterproof paper bag to maintain high commercial values under completed weed-free treatments in Japan. However, a high labour and time cost required for weeding and bagging is not worth the price of fresh tomato fruits on the market.

Regarding the weed types, the key weeds were mainly annual plants at this study site (Table 2). In the case of crop fields where perennial weeds with long rhizomes are predominant, it may be necessary to remove weeds for at least the first several years of woodchip mulching because of incomplete top covering, or a thick top covering (> 10–15 cm) may be required to control perennial weeds. Comparing organic mulches with plastic mulches, polyethene film is able to well suppress weed growth^10,47^ In particular, black polyethene film has strong effects on weed control^48^ because the light-dependent seed germination from seed banks is effectively suppressed by the dark shading^26^. However, the number of dehiscent tomato fruits may be increased by polyethene mulching, as seen in the weed-free treatment. The usefulness of woodchip mulches seems to be thus validated even in annual crops.

The maintenance of suitable soil water and nutrient contents is essential for horticultural crop production. Polyethene film provides a substantial barrier preventing water evaporation^49^, nitrogen leaching^50^ and wind erosion^51^ in soil. In terms of soil moisture, the present study showed that the extreme drop in water contents was well prevented during the prolonged periods of no precipitation in summer 2017, even with the relatively thin (3 cm) top covering in the woodchip treatment (Fig. 3f). Note that irrigation was done only once just after tomato planting. This result shows that the woodchip mulches have strong effects on suppressing excess soil evaporation, contributing to reducing watering costs. Although the competition between crops and weeds for water in their root zones is often conspicuous^52^, diminishing of watering costs is an important strategy by mulching crop fields^53^. Soil N in the second year increased significantly in only the woodchip mulch treatment (Fig. 3h, Supplementary Table 1). Woodchip mulching potentially reduces not only the amount of soil fertilization^54^ but also that of herbicides for controlling weeds^55^ in tomato crop fields. In the present study, the number of total fruits was higher in 2016 with lower soil N (a wet year) than in 2017 with higher soil N (a dry year) (Fig. 5). Thus, we found no positive effects of soil N accumulated by woodchip mulching on tomato fruit production. According to these results, we conclude that the water contents rather than N in soil was the major limiting factor for overall tomato production in this crop field. This phenomenon was likely due to fertilization operations that were repeated over a long-term period in the past because the present study was conducted in an experimental crop field of our university. Furthermore, the top dressing of fertilizer was also conducted in this experiment, to remove the effects of soil nutrients on fruit production. It is known that nutrient competition between weeds and crops intensifies in nutrient-poor conditions^11^. In our fully fertilized experiment, the effective shading by tall weeds outweighed the disadvantage of nutrient competition for tomato fruit production.

## Conclusions

Currently, we have no long-term experience with the production of annual crops using agroforestry, but the theoretical background is that organic mulches can be expected to provide various environmental advantages to agroecosystems^37^. Although some disadvantageous effects, such as lowering of soil pH by phenolic acids from decomposed wood materials, have been predicted^11^, several studies have shown no significant effects on acidity in soils even with the application of organic mulches over many years^9,56^. No allelopathic effect was found in this study either. The application of woodchip mulches for tomato cultivation contributes not only to reducing the high labour cost for hand weeding but also to removing the environmental loads from plastic and herbicide usage^16^. Since the mid-1990s, photodegradable and biodegradable polymers have been introduced in the plastic market^10^. Nevertheless, the application of these degradable polymers for mulching permits the invasion of particular perennial weeds, such as purple nutsedge (*Cyperus rotundus* L.) with long rhizome^57^. Moreover, photodegradable polymers are partially produced from fossil fuels. The replacement of plastics with natural woodchips for mulching is a definite alternative, indicating that agroforestry practices should be part of climate change mitigation and adaptation^24^. For establishing sustainable agroecosystems, more studies are needed to select adequate materials and methods for organic mulching, taking into account crop types, weed types and local social situations. Furthermore, more study of the combination and integration of woody plants and crops and their management at the landscape scale is needed for agroecological implementation.

## Materials and methods

### Study site, cultivated plants and experimental design

The crop field experiment was conducted over two successive years from 2016 to 2017 at the Center for Design-centric Engineering, Kyoto Institute of Technology, Kyoto, Japan (35° 02′ N, 135° 69′ E). The soil type in the study area is sandy brown forest soil. The F1 cultivar “Saturn”, which has a resistance against tobacco mosaic virus, was used as the scion tomato plant, and the cultivar “Helper-M”, which has a complex resistance against various insect herbivores and fungi, was used as the rootstock tomato plant. Nursery plants grafted approximately 25–27 cm high above the ground were obtained from a commercial nursery in spring of each year (Takii Co. Ltd., Kyoto, Japan). The cultivar of mulberry trees was “Minami-sakari”. The mulberry shoots pruned in winter after leaf falling in 2015 were collected, and those shoots were chopped and shredded until approximately 1–3 cm in length with a chipper shredder (CSD220-DC, Yamabiko Co. Ltd., Tokyo, Japan). The large pieces (> approximately 3 cm) were removed. The shredded woodchips were stored in mesh bags in a greenhouse and dried under air temperatures.

We set three treatments: (1) woodchip mulch, (2) weed-free (i.e., weeded) and (3) weedy (i.e., unweeded) treatments. Each treatment was conducted in three replicates (separated ridges) (Fig. 1). In the woodchip mulch treatment, the dried woodchips were ploughed into soil in April before tomato planting, and then the soil surface was covered with a woodchip thickness of approximately 3 cm. The total amount of applied woodchips was 63 ton ha^−1^.

In all treatments, no herbicides were used for weeding. To keep insects (such as cutworms and aphids) away, the granule pesticides of 3% diazinon (Diazinon 5, Nihon-Kayaku Co. Ltd., Tokyo, Japan) and those of 5% acephate (Ortran GF, Sumitomo Chemical Garden Product Inc., Tokyo, Japan) were applied to the soil surface one time just after the tomato planting in all treatments. To remove the fertilization effects of woodchip mulches on tomato fruit production, the soil nutrient levels were fully maintained at high levels in all treatments. The annual amount of compound fertilizer was 156 kg of nitrogen (N), 168 kg of phosphorate (P) and 168 kg ha^−1^ of potassium (K) in the soil in the three treatments (woodchip mulch, weed-free and weedy treatments). The annual procedure for fertilization was as follows. The amount of fertilizer applied before tomato planting was 105 kg ha^−1^, 158 kg ha^−1^ and 163 kg ha^−1^ for N, P and K, respectively; the total fertilizer applied after tomato planting (i.e., top dressing) was 6 kg ha^−1^, 10 kg ha^−1^ and 5 kg ha^−1^ for N, P and K, respectively. The top dressing was conducted at four times every two weeks. Watering was conducted only once just after tomato planting. After that, the frequency and degree of irrigation were totally dependent on natural precipitation.

In the weed-free treatment, we removed all weeds growing on the ridges by hand three times: on 14 June, 2 August and 23 August in 2016 and on 20 June, 25 July and 21 August in 2017. In all treatments, the weed density and plant identifications were simultaneously determined. These parameters were examined in all land areas in the woodchip mulch treatments, while in the weedy and weed-free treatments they were examined in a subquadrate with square of 0.6 m by 0.6 m in each ridge (i.e., three replicate in each treatment). When fruit harvesting was finished on 23 August 2016 and on 21 August 2017, all growing weeds were removed in all treatments for examination. To examine the dry mass of the aboveground parts of the removed weeds, their roots were removed by scissors. For each plant species, the aboveground parts were dried at 80 °C for 48 h and weighed.

### Tomato planting and fruit harvesting

We transplanted tomato plants at the flowering stage of the 1st flower cluster on 19 April 2016 and on 09 May 2017. The total number of planted tomato seedlings was five in 2016 and ten in 2017 in each treatment. The density of tomato plants was 1.3 m^−2^. Out of these plants, eight to 18 tomato plants in each treatment (except for the tomatoes planted at the edges of each ridge) were used to examine the tomato fruit production in each plant. To prevent plants from falling over, a prop post was stood at just beside each tomato plant at approximately 150 cm high above the ground surface. The harvesting of mature tomato fruits started in mid-June (Fig. 4). The lateral buds were removed periodically. The top bud of the apical (main) stem was thinned by hand when the 8th flower cluster grew in late August. The reason for stopping at the 8th flower cluster was as follows: it well-known that tomato fruit production decreases and the proportion of unmarketable fruits increases drastically in open field culture after the 8th cluster flowers. Mature tomato fruits were harvested at approximately three-day intervals from the 1st cluster to the 7th cluster along the apical (main) stems. To prevent crows from feeding on the tomato fruits, we enclosed the crop field with 30 mm nylon mesh bird netting during the harvesting period (June–August).

The harvested tomato fruits were separated into fresh-marketable (a) and unmarketable fruits (b–d), as shown in Fig. 2. Moreover, the unmarketable fruits were separated into the following three types: (b) dehiscent fruits, (c) fruits infected by blossom-end rot and (d) fruits that were too small (less than 115 g in fresh mass).

### Environmental measurements

The soil temperatures at 5 cm below the ground surface were measured with a thermistor sensor with a data logger (TR-7wf, T & D Co. Ltd., Nagano, Japan) in the woodchip mulch and the weed-free treatments in 2016 and in all three treatments in 2017. The soil temperatures were automatically recorded at 1-h intervals. In 2016, the seasonal change in soil water contents from 0 to 5 cm below the ground surface was periodically measured 1–4 times per month. To examine the soil water contents, the soils were collected with a stainless steel-core sampler of 100 ml (DIK-1801, Daiki Rika Kogyo Co. Ltd, Saitama, Japan). The collected soils were dried at 80 °C for 48 h and weighed. The soil water contents (%) were calculated, as follows:

1 $${\text{Soil}}\,{\text{water}}\,{\text{contents}} = \left( {\left( {{\text{wet}}\,{\text{mass }}{-}{\text{ dry}}\,{\text{mass}}} \right)/{\text{wet}}\,{\text{mass}}} \right){ }100.$$

In 2017, the soil water contents were measured with an electrical conductivity metre (DM-18, Takemura Denki Seisakujo Co. Ltd., Tokyo, Japan) in each replicate. The obtained values of electrical conductivity were calibrated to soil water contents.

To examine soil nitrogen (N) concentrations, soils from 0 to 10 cm below the ground surface were collected with a stainless steel-core sampler of 10 cm length and 4 cm diameter (DIK-106B, Daiki Rika Kogyo Co. Ltd, Saitama, Japan). Soil sampling was conducted three times a year (April, June or July and August). The soils were collected at five separate sites in each replicate, and were mixed in each replicate. The mixed soils were filtered with sieves of 8 mm mesh, 2 mm mesh and 1 mm mesh to remove stones and roots. Using 200 mg dry mass of the sieved soils, the soil N concentrations were measured with a gas chromatographer N-C analyser (JM1000CN, J-Science Lab. Co. Ltd., Kyoto, Japan).

### Statistics

All statistical analyses were conducted with the software packages of “R” (Ver. 4.1.0)^58^ and “EZR”^59^. The weeds were harvested four times a year. The weed density and dry mass, as shown in Table 1, were statistically compared among the treatments (woodchip mulch, weed-free and weedy) on each harvest day by using generalized linear mixed models (GLMMs), including random effects among three replicates in each treatment, assuming Gaussian distribution. The number of tomato fruits per plant was counted for the four fruit types: total fruits, fresh-marketable fruits, dehiscent fruits and other unmarketable fruits. In each fruit type, the number of tomato fruits per plant, as shown in Table 2, was statistically compared among the treatments by using GLMM, including random effects among three replicates in each treatment, assuming Gaussian distribution. All statistical results of the GLMM are shown in Supplementary Table 4. For soil N concentrations, the normal distribution was not statistically rejected by the Shapiro–Wilk normality test. Therefore, the soil N concentrations were statistically compared among the treatments, years (2016, 2017) and their interactive effects by two-way ANOVA, and the significant differences among the treatments in each year were examined by Sheffé's test (Supplementary Table 1). All statistical significances are recognized by *P* < 0.05. Raw data are shown in Supplementary Table 5.

### Ethical approval

All procedures in this experiment were carried out in accordance with relevant guidelines of the university field of Kyoto Institute of Technology.

## Supplementary Information

Supplementary Tables.

## Data Availability

All raw data are included in the supplementary information. Request to the corresponding authors for more detailed information.
