# Supplementary material for: Fresh-marketable tomato yields enhanced by moderate weed control and suppressed fruit dehiscence with woodchip mulching
Source: Sci Rep. 2022 Aug 2;12:13283. doi: 10.1038/s41598-022-15568-x (PMC9346129; doi:10.1038/s41598-022-15568-x)
Supplement: Supplementary file 1 — Supplementary Tables. [file 41598_2022_15568_MOESM1_ESM.pdf]

Fresh-marketable tomato yields enhanced by moderate weed control and suppressed fruit dehiscence with woodchip mulching

Sakae Horimoto, Kazuaki Fukuda, Jin Yoshimura & Atsushi Ishida

Supplementary Table 1. The mean (1 SD) values of soil nitrogen concentrations (mg g^{-1}) among three replicates in each treatment in 2016 and 2017. Different uppercase letters (A, B) show significant differences between the two years in each treatment, and different lowercase letters (a, b) show significant differences among the treatments in each year by Sheffé's test (***: $P < 0.001$, **: $P < 0.01$, *: $P < 0.05$). *F*- and *P*-values among the treatments, the years and their interactions in the results of two-way ANOVA are shown.

Year	2016	2017
Woodchip mulch	1.97 **a, A**	2.19 **a, B**
	(0.13)	(0.15)
Weed-free	1.88 **a, A**	1.87 **b, A**
	(0.14)	(0.12)
Weedy	1.88 **a, A**	1.92 **b, A**
	(0.13)	(0.13)
	F-values	*P*-values
Treatments	12.064	<0.001 ***
Years	4.8288	0.032 *
Treatments x Years	4.2690	0.020 *

Supplementary Table 2. The percentage (%) of weed plant density and weed dry mass in each plant family in the woodchip mulch and weedy treatments. These data were collected on 23 August 2016 and on 21 August 2017 (timing of the last fruit harvest).

	2016				2017			
	The number of weed plants		Dry mass of weed plants		The number of weed plants		Dry mass of weed plants	
Family	Woodchip mulch	Weedy	Woodchip mulch	Weedy	Woodchip mulch	Weedy	Woodchip mulch	Weedy
Amaranthaceae	23.8	36.9	8.1	29.9	3.0	36.2	0.1	12.8
Asteraceae	12.5	14.6	0.5	11.2	0.0	14.8	0.0	2.5
Boraginaceae	0.0	0.5	0.0	0.0	0.0	0.0	0.0	0.0
Brassicaceae	2.3	0.0	0.1	0.0	1.0	0.0	0.0	0.0
Caryophyllaceae	1.1	0.0	0.0	0.0	0.0	0.0	0.0	0.0
Commelinaceae	1.1	0.5	2.9	0.9	1.0	0.2	0.2	0.1
Convolvulaceae	1.1	0.0	0.0	0.0	0.0	0.0	0.0	0.0
Molluginaceae	1.1	0.0	0.1	0.0	0.0	0.0	0.0	0.0
Oxalidaceae	6.8	3.9	0.1	0.2	0.0	0.5	0.0	0.0
Poaceae	50.2	41.7	88.1	57.8	94.9	47.1	99.6	84.0
Portulacaceae	0.0	1.9	0.0	0.1	0.0	1.2	0.0	0.5
Total	100.0	100.0	100.0	100.0	100.0	100.0	100.0	100.0

Supplementary Table 3. Mean (1 SD) values of the plant density and dry mass per land area in each weed plant species among three replicates in the woodchip mulch and the weedy treatments. These data were collected on 23 August 2016 and on 21 August 2017 (timing of the last fruit harvest).

(a) Weeds in 2016

		2016			
Family	Species name	The number of plants per area		Dry mass of plants per area	
		m-2		g m-2	
		Woodchip mulch	Weedy	Woodchip mulch	Weedy
Amaranthaceae	*Amaranthus viridis* L.	2.1 (2.5)	45.4 (22.6)	37.47 (47.92)	102.78 (78.06)
	Chenopodium ficifolium Smith	0.2 (0.2)	25.0 (32.0)	0.66 (0.89)	113.27 (154.98)
Asteraceae	*Bidens biternata* (Lour.) Merr. et Sherff	0.6 (1.0)	0.0	0.98 (1.69)	0.00
	Eclipta prostrata (L.) L.	0.0	4.6 (1.6)	0.00	21.87 (14.47)
	Galinsoga ciliata (Raf.) Blake	0.0	20.4 (18.4)	0.00	56.58 (66.00)
	Gnaphalium pensylvanicm Willd.	0.0	0.9 (1.6)	0.00	1.49 (2.58)
	Matricaria recutita L.	0.0	0.9 (1.6)	0.00	0.34 (0.59)
	Senecio vulgaris L.	0.2 (0.4)	0.9 (1.6)	1.31 (2.27)	0.59 (1.03)
	Sonchus oleraceus L.	0.4 (0.2)	0.0	0.09 (0.07)	0.00
Boraginaceae	*Bothriospermum tenellum* (Hornem.) Fisch. et Mey.	0.0	0.9 (1.6)	0.00	0.02 (0.03)
Brassicaceae	*Capsella bursa-pastoris* (L.) Medik.	0.0	0.0	0.00	0.00
	Rorippa indica (L.) Hiern	0.2 (0.2)	0.0	0.29 (0.47)	0.00
Caryophyllaceae	*Stellaria* spp.	0.1 (0.2)	0.0	0.01 (0.02)	0.00
Commelinaceae	*Commelina communis* L.	0.1 (0.2)	0.9 (1.6)	13.60 (23.56)	6.39 (11.07)
Convolvulaceae	*Calystegia hederacea* Wall.	0.1 (0.2)	0.0	0.22 (0.37)	0.00
Molluginaceae	*Mollugo pentaphylla* L.	0.1 (0.2)	0.0	0.27 (0.46)	0.00
Oxalidaceae	*Oxalis corniculata* L.	0.7 (0.9)	7.4 (10.5)	0.57 (0.83)	1.29 (1.97)
Poaceae	*Digitaria ciliaris* (Retz.) Koeler	3.7 (1.9)	72.2 (117.9)	366.25 (55.28)	408.81 (452.07)
	Echinochloa crus-galli (L.) Beauv. var. *crus -galli*	0.1 (0.2)	1.9 (3.2)	6.41 (11.10)	7.60 (13.17)
	Eleusine indica (L.) Gaertn.	0.8 (0.8)	3.7 (4.2)	6.35 (5.87)	1.67 (2.58)
	Poa annua L.	0.0	1.9 (3.2)	0.00	0.08 (0.14)
	Setaria spp.	0.3 (0.3)	0.0	33.42 (36.97)	0.00
Portulacaceae	*Portulaca oleracea* L.	0.0	3.7 (6.4)	0.00	0.54 (0.93)
	Total	9.8 (1.1)	190.7 (104.4)	467.88 (57.74)	723.32 (592.70)

(b) Weeds in 2017

		2017			
Family	Species name	The number of plants per area		Dry mass of plants per area	
		m-2		g m-2	
		Woodchip mulch	Weedy	Woodchip mulch	Weedy
Amaranthaceae	*Amaranthus viridis* L.	0.3 (0.5)	108.6 (27.6)	0.29 (0.50)	93.04 (48.50)
	Chenopodium ficifolium Smith	0.6 (1.0)	33.3 (27.0)	0.82 (1.42)	25.04 (20.00)
Asteraceae	*Bidens biternata* (Lour.) Merr. et Sherff	0.0	0.0	0.00	0.00
	Eclipta prostrata (L.) L.	0.0	2.9 (2.9)	0.00	2.28 (2.38)
	Galinsoga ciliata (Raf.) Blake	0.0	55.2 (38.6)	0.00	21.12 (1.41)
	Gnaphalium pensylvanicm Willd.	0.0	0.0	0.00	0.00
	Matricaria recutita L.	0.0	0.0	0.00	0.00
	Senecio vulgaris L.	0.0	0.0	0.00	0.00
	Sonchus oleraceus L.	0.0	0.0	0.00	0.00
Boraginaceae	*Bothriospermum tenellum* (Hornem.) Fisch. et Mey.	0.0	0.0	0.00	0.00
Brassicaceae	*Capsella bursa-pastoris* (L.) Medik.	0.0	0.0	0.00	0.00
	Rorippa indica (L.) Hiern	0.3 (0.5)	0.0	0.16 (0.28)	0.00
Caryophyllaceae	*Stellaria* spp.	0.0	0.0	0.00	0.00
Commelinaceae	*Commelina communis* L.	0.3 (0.5)	1.0 (1.6)	2.28 (3.95)	1.28 (2.21)
Convolvulaceae	*Calystegia hederacea* Wall.	0.0	0.0	0.00	0.00
Molluginaceae	*Mollugo pentaphylla* L.	0.0	0.0	0.00	0.00
Oxalidaceae	*Oxalis corniculata* L.	0.0	1.9 (3.3)	0.00	0.11 (0.20)
Poaceae	*Digitaria ciliaris* (Retz.) Koeler	21.1 (1.7)	167.6 (119.1)	739.98 (309.04)	572.98 (245.62)
	Echinochloa crus-galli (L.) Beauv. var. *crus -galli*	0.0	3.8 (1.6)	0.00	1.51 (2.33)
	Eleusine indica (L.) Gaertn.	0.0	0.0	0.00	0.00
	Poa annua L.	0.0	0.0	0.00	0.00
	Setaria spp.	5.0 (6.6)	13.3 (20.7)	210.54 (339.59)	197.56 (339.77)
Portulacaceae	*Portulaca oleracea* L.	0.0	4.8 (4.4)	0.00	4.60 (7.60)
	Total	27.5 (8.5)	392.4 (153.4)	954.08 (114.32)	919.52 (111.81)

Supplementary Table 4. The statistical results of generalized linear mixed models (GLMMs).

(a) Weed density and the aboveground dry mass of weeds in each treatment are shown in Table 1.

Date	**14 June 2016**						**20 June 2017**				
**Weed density**					AIC		**Weed density**				AIC
	Estimate	Std. Error	*T*-value	*P*-value	116.3		Estimate	Std. Error	*T*-value	*P*-value	161.2
Woodchip * Weed-free	-318.7	19.857	-16.048	<0.001			-736.53	131.44	-5.603	<0.001	
Woodchip * Weedy	325.9	13.477	24.181	<0.001			767.63	90.45	8.487	<0.001	
Weed free * Weedy	-9.2	19.857	-0.465	0.6420			-270.47	131.44	-2.0580	0.0396	
Date	**2 August 2016**						**25 July 2017**				
**Weed density**					AIC		**Weed density**				AIC
	Estimate	Std. Error	*T*-value	*P*-value	147.7		Estimate	Std. Error	*T*-value	*P*-value	136.2
Woodchip * Weed-free	-470.73	67.89	-6.934	<0.001			-127.97	47.74	-2.681	0.0074	
Woodchip * Weedy	480.53	50.91	9.438	<0.001			102.87	47.74	2.155	0.0312	
Weed free * Weedy	-288.87	67.89	-4.255	<0.001			157.13	30.85	5.9400	<0.001	
Date	**23 August 2016**						**21 August 2017**				
**Weed density**					AIC		**Weed density**				AIC
	Estimate	Std. Error	*T*-value	*P*-value	130.4		Estimate	Std. Error	*T*-value	*P*-value	139.4
Woodchip * Weed-free	9.767	32.83	0.298	0.7660			-67.73	46.08	-1.4700	0.1416	
Woodchip * Weedy	<0.001	25.00	0.000	1.0000			95.23	37.73	2.5240	0.0116	
Weed free * Weedy	190.7	32.83	5.810	<0.001			297.17	46.08	6.4480	<0.001	
Date	**23 August 2016**				AIC		**21 August 2017**				AIC
**The aboveground dry mass in weeds**					172.3		**The aboveground dry mass in weeds**				136.6
	Estimate	Std. Error	*T*-value	*P*-value			Estimate	Std. Error	*T*-value	*P*-value	
Woodchip * Weed-free	467.9	186.3	2.511	0.0120			940.17	35.74	26.303	<0.001	
Woodchip * Weedy	<0.001	135.4	0.000	1.0000			13.93	40.92	0.341	0.733	
Weed free * Weedy	723.3	186.3	3.882	<0.001			905.57	35.74	25.335	<0.001	

(b) The number of tomato fruits per plant are shown in Table 2.

Year	**2016**						**2017**				
**The number of total fruits**					AIC		**The number of total fruits**				AIC
	Estimate	Std. Error	*T*-value	*P*-value	211.6		Estimate	Std. Error	*T*-value	*P*-value	227.8
Woodchip * Weed-free	21.6280	1.2800	16.891	<0.001			16.8571	0.8988	18.755	<0.001	
Woodchip * Weedy	1.0750	1.5460	0.695	0.4868			0.4762	1.2498	0.381	0.7032	
Weed free * Weedy	3.1780	1.7500	1.816	0.0694			2.4762	1.323	1.872	0.0613	
**The number of marketable fruits**					AIC		**The number of marketable fruits**				AIC
	Estimate	Std. Error	*T*-value	*P*-value	194.4		Estimate	Std. Error	*T*-value	*P*-value	198.4
Woodchip * Weed-free	9.3750	1.1583	8.094	<0.001			7.2857	0.6278	11.605	<0.001	
Woodchip * Weedy	-0.4861	1.3921	-0.349	0.7270			1.8476	0.8730	2.116	0.0343	
Weed free * Weedy	-1.7083	1.5919	-1.073	0.2830			-1.4524	0.9241	-1.572	0.116	
**The number of dehiscent fruits**					AIC		**The number of dehiscent fruits**				AIC
	Estimate	Std. Error	*T*-value	*P*-value	193.5		Estimate	Std. Error	*T*-value	*P*-value	209.1
Woodchip * Weed-free	8.1630	1.0630	7.679	<0.001			5.9286	0.7152	8.29	<0.001	
Woodchip * Weedy	1.0730	1.1920	0.900	0.3680			-0.1286	0.9944	-0.129	0.897	
Weed free * Weedy	5.2730	1.3510	3.903	<0.001			4.8214	1.0527	4.58	<0.001	
**The number of other unmarketable fruits**					AIC		**The number of other unmarketable fruits**				AIC
	Estimate	Std. Error	*T*-value	*P*-value	188.2		Estimate	Std. Error	*T*-value	*P*-value	162.0
Woodchip * Weed-free	4.0000	1.0597	3.775	0.0002			3.6429	0.4027	9.047	<0.001	
Woodchip * Weedy	0.5556	1.2736	0.436	0.6627			-1.2429	0.5599	-2.220	0.0264	
Weed free * Weedy	-0.3333	1.4564	-0.229	0.8190			-0.8929	0.5927	-1.506	0.1320	

Supplementary Table 5. The list of raw data obtained in this study.

(a) Soil water contents (%) in 2016.

Treatment	Ridge (Replicate)	26 April	5 May	13 May	20 May	24 May	1 June	24 June	6 July	2 August	29 August
Woodchip mulch	1	18.5	26.7	28.7	26.7	22.8	24.8	26.0	24.6	17.3	16.9
	2			28.4	28.4	23.6	27.6	27.1	21.8	20.1	13.7
	3			24.4	28.7	23.1	26.3	28.7	21.2	16.5	13.4
Weed-free	1	13.9	13.9	14.9	15.2	12.8	16.2	19.4	12.1	14.1	8.6
	2			14.2	15.3	12.4	16.8	21.0	12.0	15.3	10.4
	3			14.1	14.8	11.0	15.7	19.7	12.9	16.7	7.9
Weedy	1	13.9	13.9	14.9	15.2	12.8	16.2	21.0	13.4	14.3	7.6
	2			14.2	15.3	12.4	16.8	20.2	12.9	14.4	8.8
	3			14.1	14.8	11.0	15.7	20.6	12.8	14.4	9.1

(b) Soil water contents (%) in 2017.

Treatment	Ridge (Replicate)	9 May	19 May	5 June	14 June	20 June	27 June	13 July	27 July
Woodchip mulch	1	19.4	23.3	22.2	22.0	22.5	22.6	21.8	22.3
	1		23.6	22.5	23.0	21.8	22.6	22.5	21.6
	1		23.8	22.6	22.5	21.9	23.0	22.6	22.3
	2	16.2	23.9	22.8	22.3	21.9	23.0	22.9	22.4
	2		23.9	22.5	21.7	21.7	22.6	22.4	21.9
	2		24.2	22.3	22.2	21.5	22.8	23.1	23.0
	3	16.8	23.2	21.5	22.3	21.7	22.7	23.1	22.0
	3		23.0	22.5	22.2	21.8	22.8	23.2	22.2
	3		23.6	22.5	22.7	21.5	22.9	22.9	22.8
Weed-free	1	20.2	22.4	22.2	21.9	6	22.5	11.4	23.4
	1		22.3	21.7	21.3	0.1	22.1	18.3	21.6
	1		22.5	22.1	21.5	11.1	22.3	12.6	22.2
	2	17.3	21.9	21.6	7.8	3	22.7	19.3	22.1
	2		22.3	21.7	15.8	3.2	22.4	14.8	22.2
	2		21.9	21.9	15.6	0.3	22.7	18.2	22.1
	3	19.7	22	21.5	14.8	0.3	22.1	15.8	21.5
	3		21.5	21.6	8.5	4.3	22.6	16.9	22.7
	3		22.5	21.9	15	1.5	23.2	15.8	22.4
Weedy	1	20.2	22.1	21.4	21.7	11.6	22.4	16.8	21.6
	1		21.5	21.7	21.3	14.8	22.2	17.5	22.5
	1		22.3	22.1	22.5	21.4	22.4	17.9	22.6
	2	19	22.2	22.1	21.4	3.2	22.9	18	21.6
	2		22.2	21.8	11.9	9.7	22.1	16.4	22.1
	2		21.9	21.5	9.4	17.9	22.5	18.1	21.9
	3	18.5	21.7	21.3	21.4	6	22.5	18.8	22.4
	3		22.3	22.4	15	6	22.6	17.8	22.6
	3		22.2	21.4	16	6.8	22.5	16.5	22.1

(c) Soil nitrogen concentration (mg g^{-1}).

	Year	2016	2016	2016		2017	2017	2017
Treatment	Ridge (Replicate)	15 April	22 June	19 August		13 April	27 July	23 August
Woodchip mulch	1	1.74	1.82	1.91		2.00	2.00	2.10
	2	1.97	2.01	2.00		2.30	2.10	2.20
	3	1.98	2.13	2.16		2.40	2.30	2.30
Weed-free	1	1.68	1.76	1.75		1.70	1.70	1.80
	2	1.96	2.02	2.08		2.00	1.80	2.00
	3	1.82	1.97	1.89		1.90	2.00	1.90
Weedy	1	1.68	1.79	1.77		1.70	1.80	1.80
	2	1.96	2.06	2.03		2.10	2.00	2.00
	3	1.82	1.85	1.93		2.00	1.90	2.00

(d) The total number of harvested tomato fruits per plant.

Year		2016						2017			
Treatment	Ridge (Replicate)	No. of total fruits	No. of marketable fruits	No. of dehiscent fruits	No. of other unmarketable fruits		Ridge (Replicate)	No. of total fruits	No. of marketable fruits	No. of dehiscent fruits	No. of other unmarketable fruits
Woodchip mulch	1	27	11	8	8		1	23	14	4	5
	1	24	10	2	12		1	19	6	11	2
	1	25	8	14	3		1	18	9	7	2
	2	21	10	7	4		1	21	11	8	2
	2	22	10	7	5		1	21	12	6	3
	2	18	6	8	4		2	20	11	6	3
	2	15	3	11	1		2	13	6	4	3
	2	23	7	12	4		2	15	4	8	3
	2	22	13	5	4		2	15	4	6	5
	2	27	9	7	11		2	13	8	3	2
	2	23	15	7	1		3	15	11	3	1
	3	32	11	9	12		3	17	10	6	1
	3	23	5	11	7		3	12	8	3	1
	3	23	8	15	0		3	19	10	7	2
	3	23	8	13	2		3	19	13	5	1
	3	19	4	12	3						
	3	23	16	7	0						
	3	18	6	11	1						
Weed-free	1	25	8	15	2		1	19	4	12	3
	1	22	2	15	5		1	15	4	7	4
	1	25	10	13	2		2	18	7	9	2
	2	19	5	10	4		2	14	7	7	0
	2	26	9	13	4		2	18	4	12	2
	2	24	5	12	7		2	23	4	13	6
	3	23	11	8	4		2	25	9	10	6
	3	29	6	20	3		3	19	6	10	3
	3	30	13	15	2		3	17	8	7	2
							3	16	2	13	1
							3	20	8	10	2
							3	28	7	19	2
Weedy	1	15	6	8	1		1	16	5	9	2
	1	19	8	5	6		1	15	10	1	4
	1	28	10	11	7		1	15	7	4	4
	2	21	10	8	3		1	17	7	6	4
	2	19	7	7	5		2	22	10	8	4
	3	22	8	9	5		2	17	9	7	1
	3	29	16	11	2		2	17	8	4	5
	3	20	10	7	3		2	18	7	5	6
							2	24	9	12	3
							3	16	6	7	3
							3	15	6	4	5
							3	17	7	4	6
							3	15	7	7	1
							3	12	4	5	3

(e) Weed density (m^{-2}) and the aboveground dry mass ($g\ m^{-2}$) of weeds.

Year		2016				2017			
Date		14 June	02 August	23 August	23 August	20 June	25 July	21 August	21 August
Treatment	Ridge (Replicate)	Weed density			Weed dry mass	Weed density			Weed dry mass
Wood chip mulch	1	7.0	9.0	9.3	412.0	28.3	34.2	20.0	823.1
	2	6.7	10.7	11	464.2	38.3	28.3	36.7	1005.6
	3	8.0	9.7	9	527.4	26.7	25.0	25.8	1033.6
Weed-free	1	350.0	697.2	0	0	1100.0	251.4	88.6	11.9
	2	319.4	275.0	0	0	562.9	88.6	80.0	11.4
	3	308.3	469.4	0	0	640.0	131.4	117.1	18.5
Weedy	1	294.4	169.4	311.1	1392.2	374.3	168.6	320.0	792.4
	2	277.8	177.8	125.0	263.3	348.6	237.1	288.6	1002.7
	3	377.8	227.8	136.1	514.5	768.6	374.3	568.6	963.4
